# When the technical is also normative: a critical assessment of measuring health inequalities using the concentration index-based indices

**DOI:** 10.1186/s12963-022-00299-y

**Published:** 2022-12-01

**Authors:** Paul Contoyannis, Jeremiah Hurley, Marjan Walli-Attaei

**Affiliations:** 1 Department of Economics and the Center for Health Economics and Policy Analysis, McMaster University, Hamilton, ON Canada; 2 Population Health Research Institute, Hamilton Health Sciences and McMaster University, Hamilton, ON L8L 2X2 Canada

**Keywords:** Measurement of inequality, Income-related health inequality, Concentration index, Wagstaff index, Erreygers index, Normative values

## Abstract

**Background:**

Concentration index-based measures are one of the most popular tools for estimating socioeconomic-status-related health inequalities. In recent years, several variants of the concentration index have been developed that are designed to correct for deficiencies of the standard concentration index and which are increasingly being used. These variants, which include the Wagstaff index and the Erreygers index, have important technical and normative differences.

**Main body:**

In this study, we provide a non-technical review and critical assessment of these indices. We (i) discuss the difficulties that arise when measurement tools intended for income are applied in a health context, (ii) describe and illustrate the interrelationship between the technical and normative properties of these indices, (iii) discuss challenges that arise when determining whether index estimates are large or of policy significance, and (iv) evaluate the alignment of research practice with the properties of the indices used. Issues discussed in parts (i) and (ii) include the different conceptions of inequality that underpin the indices, the types of changes to a distribution which leave inequality unchanged and the importance of the measurement scale and range of the outcome variable. These concepts are illustrated using hypothetical examples. For parts (iii) and (iv), we reviewed 44 empirical studies published between 2015 and 2017 and find that researchers often fail to provide meaningful interpretations of the index estimates.

**Conclusion:**

We propose a series of questions to facilitate further sensitivity analyses and provide a better understanding of the index estimates. We also provide a guide for researchers and policy analysts to facilitate the critical assessment of studies using these indices, while helping applied researchers to choose inequality measures that have the normative properties they seek.

**Supplementary Information:**

The online version contains supplementary material available at 10.1186/s12963-022-00299-y.

## Background

The concentration index (CI) is an increasingly popular measure of socioeconomic-related (SES) health inequalities. The CI’s popularity is partly attributable to its many desirable properties, such as its decomposability into factors contributing to observed inequality, its use of information from the full distributions of health and income, and its amenability for statistical inference (see, e.g., O’Donnell et al. [1]). But the CI is not without controversy. Originally developed in the context of measuring *income* inequality [2], some argue that the CI has problematic properties as a measure of SES-related *health* inequality. The CI, for example, assumes that health is measured on an unbounded, ratio scale and that a transfer of health from the rich to the poor is desirable. Controversy regarding properties of the CI has spawned the development of several closely related alternatives to the CI; these include the extended concentration index [3], generalized concentration index [4], generalized extended concentration index [5], symmetric index [5], Erreygers index [6], and Wagstaff index [7]. We will call these the CI-based indices.

This controversy and the associated proliferation of the CI-based indices are rooted in the intertwining of technical properties and normative assumptions in inequality measurement such that what may appear to be purely technical matters have (often) underappreciated normative implications. These normative implications pertain, for instance, to what constitutes inequality or how much one cares about health inequality at different parts of the income distribution. For non-specialists who simply seek to apply a suitable measure of inequality or to interpret the current evidence on the nature and extent of socioeconomic-related health inequalities, these debates can be arcane, confusing, and inaccessible both technically and because they are spread among disparate journals. Understanding the issues and options for measuring socioeconomic-related health inequality is important: different CI-based indices can lead to different empirical conclusions regarding the extent of inequality, whether inequalities differ across jurisdictions, and whether inequalities have increased or decreased over time within a jurisdiction. Further, such understanding can help practitioners choose inequality measures more consistent with the underlying values of the setting in which inequality is being measured.

This paper examines in a non-technical way many of the core issues in the debate about alternative CI-based measures of socioeconomic-related health inequality, with the goals of helping policy researchers and policy-analysts be more critical consumers of this literature and helping applied inequality researchers choose inequality measures that embody the normative properties they seek. We examine ways in which health differs from income that matter for the measurement of SES-related health inequality and highlight how describing, estimating, and evaluating health inequalities are intrinsically technical and value-laden exercises. We also discuss the difficulties that arise when interpreting the index estimates and their policy significance. Throughout the paper, we contextualize our arguments using the results from a review of empirical studies between 2015 and 2017 that apply the CI-based indices to obtain estimates of SES-related health inequality (see Additional file 1: Table S1). This review of research practice serves to empirically illustrate how well research practice aligns with the properties of the indices used. By making the linkages between normative judgments on fairness and their manifestation in the technical measurement *process* explicit, we clarify the type of information the CI-based indices provide, when they are appropriate, and their limitations.

## The concentration index-based indices

The CI is a bivariate inequality index: It measures how variation in one variable (e.g., health) relates to variation in a second variable (e.g., socioeconomic status). The CI derives from the concentration curve. A *concentration curve* plots the cumulative shares of a health variable (vertical axis) against cumulative shares of the population, ranked by an indicator of socioeconomic status (horizontal axis) [1]. Most analyses use income as the measure of socioeconomic status, but other measures can be used such as social class, educational attainment, and consumption. How best to measure socioeconomic status is much debated [8, 9] and depends importantly on availability and quality of data (e.g., asset-based approaches may be more appropriate where good income or wealth data are not available). Other issues are specific to the application of CI-based approaches; for instance, CI indices can be biased when socioeconomic status is summarized with fewer categories than the actual number of categories (e.g., grouping income into deciles leads to bias but using education in terms of obtained degree will not lead to bias because the variable itself is categorical) [10]. The choice of a SES measure deserves careful consideration but is beyond the focus of this paper. We assume one has a valid measure of socioeconomic status by which to rank individuals.

**Fig. 1:**
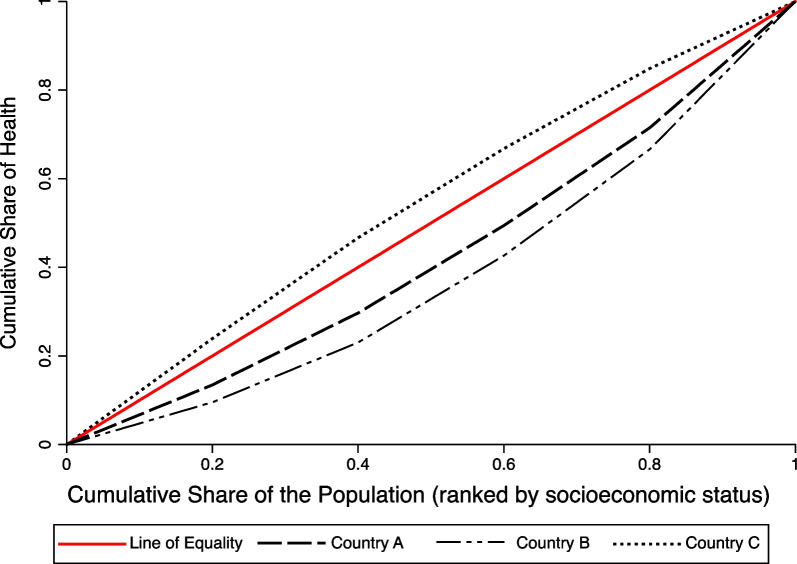
Concentration curves for three hypothetical populations

Figure 1 displays concentration curves for the populations of three hypothetical countries, A, B, and C. The concentration curve lies on the 45° line—the line of equality—in two situations: if everyone in a population has the same value of health, so that health is distributed perfectly equally, and when health variation is present but it is not associated with SES so there is no SES-related inequality. Any deviation from the 45° line indicates SES-related inequality, and the amount of inequality can be measured as a function of the deviation between the line of equality and the observed concentration curve across the income distribution. In particular, the CI, hereafter referred to as the standard CI, is equal to twice the area between a concentration curve and the line of equality, and, under conditions we discuss below, it takes on values from − 1 to + 1. The value of the standard CI is zero when the concentration curve lies on the line of equality. By convention, the standard CI and the CI-based indices more generally are positive when a concentration curve lies below the line of equality, such as countries A and B in Fig. 1, implying that higher socioeconomic groups have better health (often referred to as a “pro-rich” distribution) and are negative when it lies above the line of equality, such as country C, implying that lower socioeconomic groups have better health (often referred to as a “pro-poor” distribution). Concentration curves can cross the line of equality. If the areas above and below cancel each other out, CI-based indices can again take on the value of zero [1]. But perhaps more importantly, when concentration curves for different countries cross, different (relative) CI-based indices can rank the countries differently with respect to the amount of socioeconomic-related health inequality.^1^ Such ranking differences arise because different indices weigh observations differently (an issue we will discuss in more detail below), and when concentration curves cross, the ranking will depend in part on where they cross.

All CI-based indices are rank-dependent measures of inequality. The defining characteristic of a rank-dependent index is that calculation of the index value requires ranking the population from lowest to highest with respect to a characteristic of interest—in this context, socioeconomic status. (Recall that the population in Fig. 1 is ranked from lowest to highest socioeconomic status.) All rank-dependent measures of health inequality, including all CI-based measures, take the following generic form [11]:

1 $$I\left( h \right) = f\left( {\mu_{h} ,n,a_{h} ,b_{h} } \right)\mathop \sum \limits_{i = 1}^{n} z_{i} h_{i} .$$

where *n* represents the population size, *i* represents a single individual in the population, *μ*_h_ represents average health in the population, *b*_h_ represents the upper bound of the health variable, *a*_h_ represents the lower bound of the health variable, $f\left( {\mu_{{\text{h}}} ,n, a_{{\text{h}}} ,b_{{\text{h}}} } \right)$ is a rescaling or normalization function, and $\sum\nolimits_{i = 1}^{n} {z_{i} } h_{i}$ is a weighted sum of the health measure *h*_*i*_ with weight *z*_*i*_ for individual *i*. Hence, *all* rank-dependent indices are weighted averages of the health variable of interest: Different indices simply specify different weights (*z*_*i*_) and/or normalization functions (*f*(·)). Differences in the weights (*z*) affect the relative contribution of each observation to the index, while differences in normalization factor (*f*(·)) affect the absolute contributions of the observations to the index, depending on the mean level of health, the size of the population, and, for bounded health variables, the lower (*a*_h_) and upper (*b*_h_). bounds. These different weighting and normalization functions embed normative assumptions that generate controversy. The standard CI, for example, takes the following form:

2 $$C\left( h \right) = \frac{2}{{n^{2} \mu_{{\text{h}}} }}\mathop \sum \limits_{i = 1}^{n} z_{i} h_{i},$$

while the Erreygers index is specified as:

3 $$E\left( h \right) = \frac{8}{{n^{2} \left( {b_{{\text{h}}} - a_{{\text{h}}} } \right)}}\mathop \sum \limits_{i = 1}^{n} z_{i} h_{i}$$

The only difference between (ii) and (iii) is the normalization function, though even small differences in the normalization function can have important implications for inequality measurement, such as which distribution of the outcome constitutes the maximum amount of SES-related health inequality in society (i.e., the most unequal society).

**Fig. 2:**
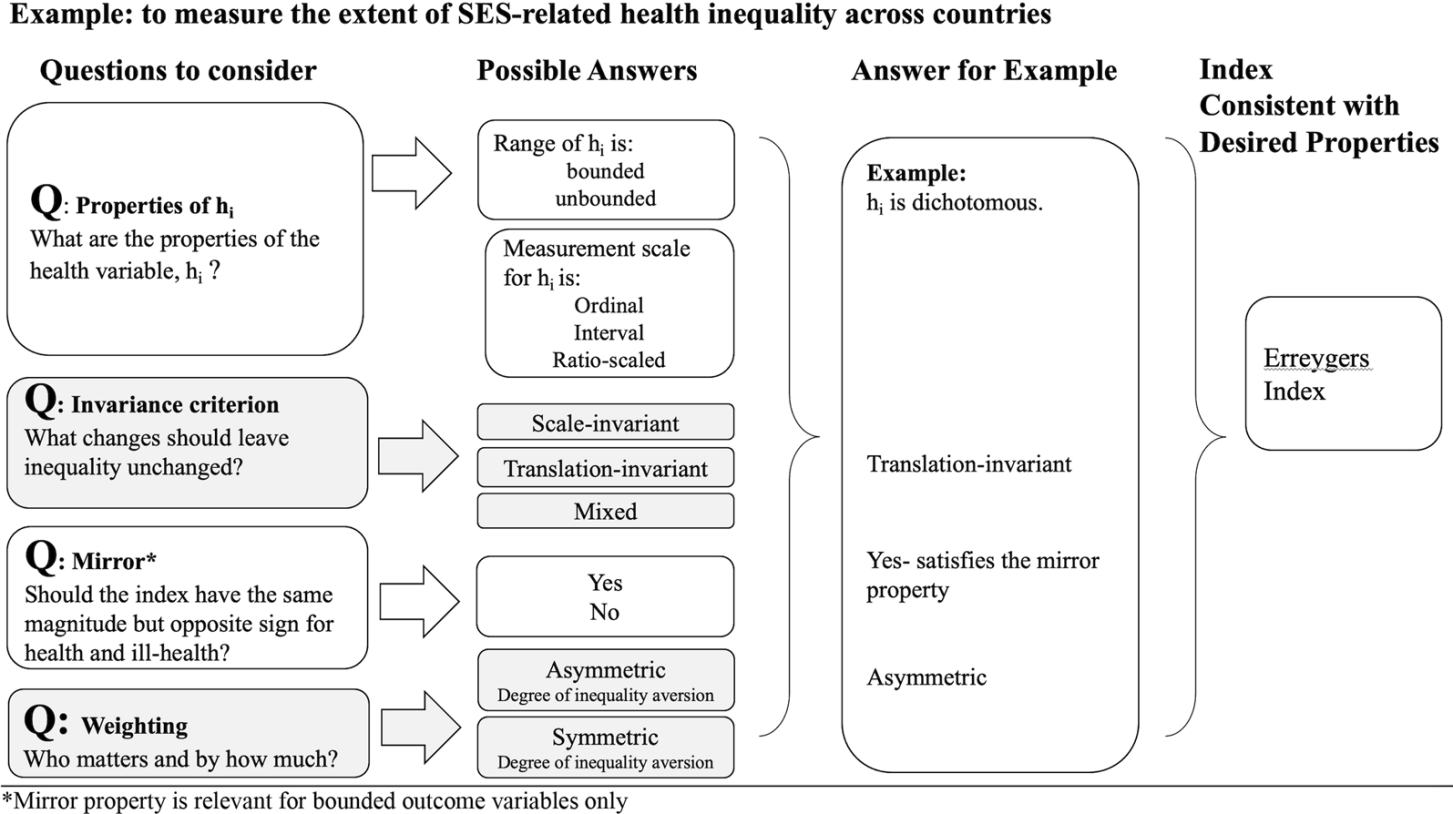
A flowchart of questions for choosing among the CI-based indices

In Table 1, we summarize the CI-based indices in terms of their technical and normative assumptions. In Fig. 2, we present a flowchart of critical questions that can be used as a guide in choosing among the CI-based indices. Sections 3.0 and 4.0 of the paper are organized around these questions. In Sect. 5.0, we discuss the difficulties that arise when interpreting the index estimates and their policy significance. Section 6.0 concludes.

**Table 1:** Summary of CI-based index properties and their health variable requirements

	Properties of the health variable (*h*_i_)				Properties of the index							
	Interval^b^	Ratio	Unbounded	Bounded	Absolute	Relative	Mixed	Quasi-absolute	Mirror	Transfer	Weighting scheme	Index equation^a^
Standard CI		✓	✓			✓				✓	Fixed. Inequality aversion parameter (*v*) = 2	$\frac{1}{n}\mathop \sum \limits_{i = 1}^{n} \left\{ {\frac{{h_{i} }}{{\overline{h}}}\left( {2R_{i} - 1} \right)} \right\}$
Modified CI	✓		✓			✓				✓	Inequality aversion parameter (*v*) = 2	$\frac{1}{n}\mathop \sum \limits_{i = 1}^{n} \left\{ {\frac{{h_{i} }}{{\overline{h} - a_{h} }}\left( {2R_{i} - 1} \right)} \right\}$
Generalized CI	✓	✓	✓		✓				✓	✓	Inequality aversion parameter (*v*) = 2	$\frac{1}{n}\mathop \sum \limits_{i = 1}^{n} \left\{ {h_{i} \left( {2R_{i} - 1} \right)} \right\}$
Extended CI		✓	✓			✓				✓	Asymmetric Inequality aversion parameter (*v*) can be varied	$\frac{1}{n}\mathop \sum \limits_{i = 1}^{n} \left[ {\frac{{h_{i} }}{{\overline{h}}} \left\{ {1 - v\left( {1 - R_{i} } \right)^{v - 1} } \right\}} \right]$
Generalized extended CI	✓	✓	✓	✓	✓				✓	✓	Inequality aversion parameter (*v*) can be varied	$\frac{1}{n}\mathop \sum \limits_{i = 1}^{n} \left[ {\left( {\frac{{v^{{\frac{v}{v - 1}}} }}{v - 1}} \right)\left( {\frac{{h_{i} - a^{h} }}{{b_{h} - a_{h} }}} \right)\left\{ {1 - v\left( {1 - R_{i} } \right)^{v - 1} } \right\}} \right]$
Wagstaff index	✓	✓		✓			✓		✓	✓	Fixed	$\frac{1}{n}\mathop \sum \limits_{i = 1}^{n} \left[ {h_{i} \left\{ {\frac{{h_{i} \left( {b_{h} - a_{h} } \right)}}{{\left( {b_{{\text{h}}} - \overline{h}} \right)\left( {\overline{h} - a_{{\text{h}}} } \right)}}} \right\}\left( {2R_{i} - 1} \right)} \right]$
Erreygers index	✓	✓		✓b				✓	✓	✓	Fixed	$\frac{1}{n}\mathop \sum \limits_{i = 1}^{n} \left\{ {4\frac{{h_{i} }}{{\left( {b_{h} - a_{{\text{h}}} } \right)}}\left( {2R_{i} - 1} \right)} \right\}$
Symmetric index		✓	✓			✓				✓	Symmetric Inequality aversion parameter (*β*) can be varied *β* can be varied	$\frac{1}{n}\mathop \sum \limits_{i = 1}^{n} \left( {\frac{{h_{i} }}{{\overline{h}}}} \right)\left[ {\beta 2^{\beta - 2} \left\{ {\left( {R_{i} - \frac{1}{2}} \right)^{2} } \right\}^{{\frac{\beta - 2}{2}}} \left( {R_{i} - \frac{1}{2}} \right)} \right]$
Generalized symmetric index	✓	✓	✓	✓	✓				✓	✓	Symmetric Inequality aversion parameter (*β*) can be varied *β* can be varied	$\frac{1}{n}\mathop \sum \limits_{i = 1}^{n} 4\left( {\frac{{h_{i} - a^{{\text{h}}} }}{{b_{{\text{h}}} - a_{{\text{h}}} }}} \right)\left[ {\beta 2^{\beta - 2} \left\{ {\left( {R_{i} - \frac{1}{2}} \right)^{2} } \right\}^{{\frac{\beta - 2}{2}}} \left( {R_{i} - \frac{1}{2}} \right)} \right]$

^a^*R*_*i*_ is the rank of the *i*th person in the SES distribution and $\overline{h}$ represents the sample mean. ^b^Except for Erreygers index where measurement scale does not matter, *h*_i_ should be measured in same unit to ensure differences in estimates are not reflective of arbitrary differences in measurement unit, i.e., weight measured in same units (kg) for all observations

## Health measures and the CI-based indices

Every CI-based inequality index assumes that the health measure (e.g., life years gained, number of physician visits, presence or absence of disease) satisfies certain measurement properties. An estimate of inequality is valid only to the extent that measurement properties assumed by the chosen index match those of the health measure. Although the standard CI is widely used in health inequality measurement, it was originally developed to measure aspects of income inequality, and health measures often have properties quite distinct from those of income and other monetary measures. We focus on two measurement properties: the *range* and the *measurement scale* of the outcome measure.

Income has an unbounded, positive range; in principle, it can take on infinitely large positive values. Unlike income, however, the range of values a health measure can take is often strictly bounded; indeed, health measures are often doubly bounded, with fixed lower and upper values. Dichotomous measures indicating the presence or absence of a condition (low birth-weight, diabetes, death, etc.), for example, can take on one of only two values (0 = not present; 1 = present); measures of health-related quality of life often have a defined range (e.g., 0 to 1). Even measures such as life expectancy are, many argue, doubly bounded by 0 (birth) and a biological maximum length of life (e.g., Dong et al. [12]). For a doubly bounded health measure, health can be measured either by health attainment (e.g., health-related quality of life; life expectancy) or by health shortfall (e.g., deviation from full health, life years lost), and we observe both in the literature.

**Fig. 3:**
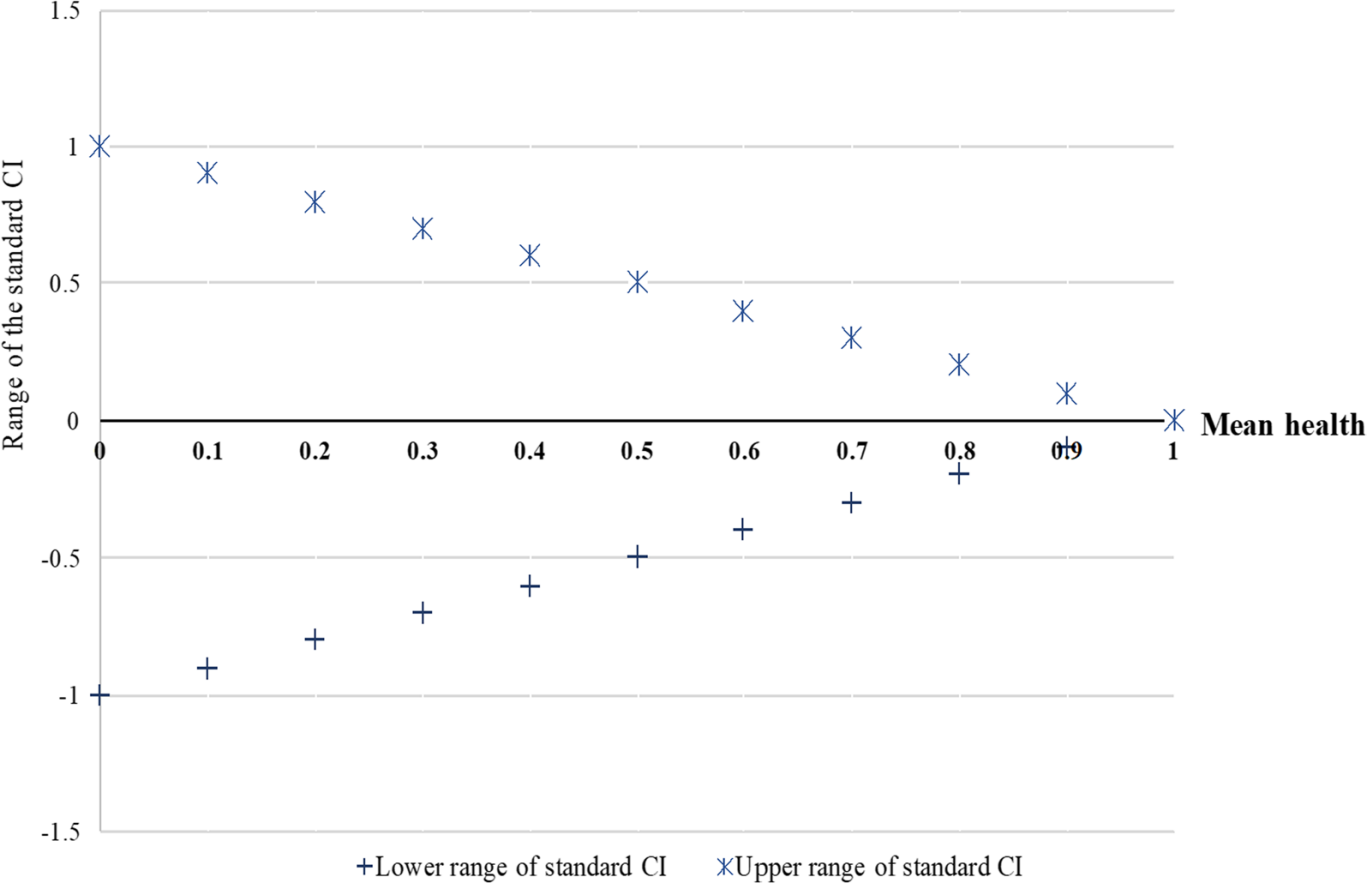
The lower and upper bounds of the standard CI for a dichotomous (0, 1) outcome variable. *Note*: Adapted from Wagstaff [7]

When the health measure is bounded, the range of possible values for some CI-based indices, such as the standard CI, depends on the mean of the health variable in the population [6, 7, 13]. In such cases, as average health in a population increases, the range of possible values for the standard CI narrows (see Fig. 3) [7]. In these situations, an analyst using the standard CI implicitly agrees that relative inequality is smaller the larger the mean, or in other words, that relative health differences are smaller the larger the mean health. If the analyst instead believes that maximally attainable inequality leads to the same welfare loss for any level of mean health, the Wagstaff index—which has a fixed range—is a more appropriate index. Our review of empirical studies revealed that the vast majority of the studies (40 out of 42) used bounded health measures. Twenty of these 40 studies acknowledged that the range of the index may vary when the outcome is bounded, and 2 of these 40 presented the estimates in relation to the minimum and maximum range of the index. This may matter for the interpretation of index estimates: if the means of bounded health variables differ notably, the data-based range of the CI-based indices can be notably different from the theoretical range of − 1 to + 1.

The measurement scale of a health outcome reflects the quantitative information embedded in the measure and determines the mathematical transformations that can *legitimately* be applied to it. Health is generally measured on ordinal, interval, or ratio scales. Health measured on an ordinal scale, such as self-assessed health, indicates only whether health is greater or less than some reference point, but does not provide any quantitative information about how much greater or less. That is, we know that someone who reports that they are now in excellent health rates their health as better than previously when they said they were in poor health, but we do not know how much better. Given this limited quantitative information, strictly speaking, it is not valid to calculate averages or differences for health variables measured on an ordinal scale. For health measured on an interval scale, such as the Health Utilities Index [14], which fix the zero point by convention (0 = dead), differences in values have quantitative meaning, so addition and subtraction are valid but multiplication and division are not. Finally, for health measured on a ratio scale for which there is a naturally defined zero point, such as weight or life expectancy, all arithmetic operations are valid. As with the range, the measurement scale is important because different inequality indices make differing assumptions about the measurement scale for the outcome of interest. The standard CI, for instance, assumes that the outcome is measured on a ratio scale.

Because the measurement scales of typical income measures are unbounded and ratio scaled, discussion of these issues has not figured prominently in the literature on income inequality and little thought was given to them when the standard CI was first used in the field of health inequality measurement. But as applications of the standard CI to health inequality measurement grew, often applied to health measures with different measurement scales, analysts recognized the incompatibility between the properties of some health measures and the requirements of the standard CI [6, 7, 13]. In our review of empirical studies using the CI-based indices, only 9 of 44 studies reviewed used a ratio-scaled health measure. For most of the studies, the health measure was dichotomous: 40 out of 44 studies. (Some studies had more than one health measure.) The measurement scale was unclear in 2 of the 44 studies. Many of the newly developed variants of the standard CI, such as the modified CI, the generalized CI, the Wagstaff index, and the Erreygers index attempt to relax these assumptions about the measurement properties of outcome variables, making them suitable for a wider set of health outcome measures.

## CI-based inequality indices and implicit definitions of inequality

Everyone can agree on the presence or absence of inequality: Inequality is present when the observations are not all equal. But quantifying *the degree* of inequality among differing unequal distributions is both challenging and subject to considerable disagreement about the differences in distributions that constitute greater or lesser inequality. Unavoidably, every inequality index embeds specific, often implicit, assumptions about which aspects of a distribution affect the measured degree of inequality. We consider three assumptions embedded in every index: (a) the types of changes to an *entire* distribution which leave inequality unchanged; (b) types of changes to *parts* of a distribution which leave inequality unchanged, (c) the characterization of the most unequal distribution of health in a society. To further elucidate these concepts, Table 2 illustrates how the different indices respond to these types of changes using a hypothetical example with a population of five people. The population is ranked from lowest to highest income, and life expectancy is used as the measure of health. Income-related health inequality is calculated using six indices: the standard CI, the extended CI, the generalized CI, the symmetric index, the Wagstaff index, and the Erreygers index.

**Table 2:** Estimates of income-related health inequality for the CI-based indices under various changes to the health distribution

Individuals (*i*) *n* = 5	Income (dollars)	(1) Baseline life expectancy (LE)	(2) 5% increase in LE	(3) Increase of 5 yrs to LE	(4) Ill-health (assume max LE of 120)	(5) Transfer of LE
1	20,000	59	61.95	64	61	59 + 3 → 62
2	30,000	63	66.15	68	57	63
3	58,000	70	73.50	75	50	70
4	74,000	85	89.25	90	35	85
5	100,000	92	96.60	97	28	92.3 → 89
Mean	56,400	73.80	77.49	78.80	46.20	73.80
Standard CI						
Point estimate		0.095	0.095	0.089	− 0.152	0.082
Generalized CI						
Point estimate		7.040	7.392	7.040	− 7.04	6.080
Extended CI, with inequality aversion = 3		0.139	0.139	0.130	− 0.221	0.119
Symmetric index, with inequality aversion = 3		0.103	0.103	0.097	− 0.165	0.087
Wagstaff index						
Point estimate × assume max LE of 120 yrs		0.248	0.269	0.260	− 0.248	0.214
Erreygers index						
Point estimate × assume max LE of 120 yrs		0.235	0.246	0.235	− 0.235	0.203

The Wagstaff and Erreygers indices can only be used with bounded health variables

Point estimates were calculated using “conindex” STATA package. Numbers rounded to three decimal places

### Uniform changes to the entire distribution of health: relative versus absolute indices

Let I(*h*) represent an index of inequality, where *h* refers to the full distribution of health in a population and *h*_*i*_ to the health of individual *i.* Suppose everyone’s health increases proportionately by 5 percent, so that *h*_i_(after) = 1.05 × *h*_i_(before) for everyone (i.e., all *i*). Has inequality changed? A *scale-invariant* index, such as the standard CI, says no, inequality has not changed, so I(*h*(after)) = I(*h*(before)). In Table 2, for example, note that the standard CI for distributions 1 and 2 are the same (0.095). Scale invariance means that uniform, equiproportionate changes to an entire distribution leave measured inequality unchanged. Scale invariance is valuable in some contexts. For multi-country comparisons of an outcome measured in monetary units, such as health expenditures, scale invariance means that the estimated amount of inequality is the same regardless of which jurisdiction’s currency is chosen as the common currency.

A second type of uniform change to a distribution arises when everyone’s health changes by the same absolute amount: *h*_i_(after) = *h*_i_(before) + *x*, where *x* is the absolute difference in health for everyone. Again, we can ask, has inequality changed? A *translation invariant* index, such as the generalized CI, says no: Note that the value of the generalized CI equals 7.04 for both distributions 1 and 3 after a 5-year increase in life expectancy for everyone. Translation invariance means that uniform, absolute changes to an entire distribution leave measured inequality unchanged. Note, however, that the value of the standard CI, which is a scale invariant index, differs for distributions 1 and 3 (0.095 and 0.089). This is because scale invariance focuses on ratios, while translation invariance focuses on differences.

While scale and translation invariance are commonly presented as technical assumptions, whether uniform relative or absolute changes to a distribution leave inequality unchanged is a normative matter. The normative implications of scale invariance and translation invariance have been discussed both in the health inequality literature (see, for example, Wagstaff et al. [15] Asada [16]; Harper et al. [17]; Kjellsson et al. [18]; Wagstaff [19]) and the income inequality literature (see, for example, Kolm [20]; Subramanian [21]). The income inequality literature generally favors relative measures of inequality, and in fact, absolute measures of income inequality are seldom used [22]. This is partly because scale invariance allows for real (rather than nominal) comparisons across space and time [21, 22]. Absolute measures of income inequality create complications arising from the conversion from monetary to real quantities. Within the health inequality literature, some (e.g., Atkinson [23]; Fleurbaey and Schokkaert [24]; Mackenbach [25]) favor absolute measures of inequality. Fleurbaey and Schokkaert [24], for example, argue that the absolute measurement approach aligns with equity frameworks derived from the literature on responsibility and compensation. Others, such as Wagstaff [19], have argued that reductions in absolute inequality are less common in practice and more difficult to achieve compared to reductions in relative inequalities. This difficulty in reducing absolute inequalities arises because a much larger percentage change is required for those with initial lower levels of health than for those with initial higher levels of health. In Table 2, an increment of 5 years for everyone (distribution 3) is equivalent to an 8% increase in life expectancy for quintiles 1 and 2 but only a 5% increase for quintile 5.

We have limited evidence regarding how people judge the impact of absolute versus relative changes on inequality. In the context of income inequality, the limited empirical evidence indicates that people differ in their views on inequality. In one study, a third of respondents made choices consistent with scale invariance, a sixth of the respondents made choices consistent with translation invariance [26], and the rest indicated either that their perception of inequality depends on the income levels or that none of the options presented appealed to them. A separate study found that a third of the respondents made choices consistent with scale invariance and 22% made choices consistent with translation invariance [27]. There is no evidence, to our knowledge, about such judgments in the context of health inequality. Hence, to the extent that we want to use indices that match societal preferences, we have little empirical basis for choosing between a relative or absolute inequality index.

#### Indices for bounded health variables

Many health variables of interest are bounded, and so increasingly the Wagstaff and Erreygers indices are being used alongside the standard CI for the evaluation of SES-related health inequality. In our review of empirical papers, 21 out of the 44 studies used either the Wagstaff index, the Erreygers index or both in their assessment of SES-related health inequality. When the health variable is bounded, uniform changes to the entire health distribution may not be feasible. For a health variable defined over the interval [0,1] with at least some values greater than 0.5, it is not possible to have uniform changes of either 100% or of 0.5 absolute units because such changes would extend health values outside the allowable interval of [0,1]. Hence, the Wagstaff and Erreygers indices, which were introduced specifically for bounded health variables, are neither absolute nor relative in the traditional sense because they do not strictly satisfy the properties of scale invariance or translation invariance [11, 13].^2^

The Wagstaff index was initially proposed as a correction to the standard CI to deal with issues related to the range of the standard CI when the health variable is bounded [7]. Wagstaff’s correction divides the standard CI by its upper bound, and the range of the resulting index always extends from − 1 to + 1. Wagstaff’s index is the difference of the standard CI of health attainment and health shortfall [11]. For example, in Table 2, if for the standard CI we subtract shortfall inequality from attainment inequality (attainment inequality, i.e., standard CI = 0.095; shortfall inequality, i.e., standard CI = −0.152), we obtain the estimate observed for the Wagstaff index (0.248). The invariance criteria underlying the Wagstaff index—the types of changes to the health distribution that leave measured inequality unchanged—depend on mean health in the distribution [28]. Further, because of these differences in invariance assumptions, the Wagstaff index behaves differently to the standard CI under various transformations of the health distribution. Table 2 illustrates, for example, an equiproportionate increase of 5% in life expectancy causes an increase in the Wagstaff index, while estimates of income-related health inequality remain constant for the standard CI; an equal increment of 5 years in life expectancy causes an increase in estimates of income-related health inequality for the Wagstaff index but a reduction for the standard CI.

Erreygers’ index is translation invariant for feasible uniform changes: Equal increments to the health distribution leave measured inequality unchanged. The Erreygers index is related to the generalized CI through the following rescaling factor: (4/*b*_h_ − *a*_h_), where *b*_h_ is the upper bound of the health variable and *a*_h_ is the lower bound. Hence, this rescaling factor takes into consideration the bounds of the health variable. To illustrate the relationship between the Erreygers index and the generalized CI, observe how the point estimate for the Erreygers index is equal to (4/*b*_h_ − *a*_h_) × GCI in distribution 4 of Table 2.

Referring to the Wagstaff and Erreygers indices as “corrected” CI is inaccurate. They do not “correct” the standard CI but rather relax assumptions to accommodate non-ratio scaled and bounded health variables. These indices have their own unique properties and normative assumptions that differ from the standard CI including, as already noted, the types of changes to the health distribution that leave the index value unchanged, the types of changes that reduce SES-related health inequalities, and the definition of the most unequal society. For the standard CI, the extended CI, and the generalized CI, for example, maximum SES-related inequality occurs when either the richest *person* has positive health and all others have “zero” health or the poorest person has positive health and all others have “zero” health. For the Wagstaff index, the maximum SES-related inequality arises when a given richest *proportion* of the population has all the health, where this proportion depends on mean health in the population; for the Erreygers index the maximum SES-related inequality arises when the richest 50% have *all* the health [11]. Hence, the variants of the standard CI can represent different conceptions of the very nature of inequality in a population.

Our review of the empirical papers found that several studies employing the Wagstaff and/or Erreygers indices refer to these alternative indices as corrections to the standard CI (e.g., Cabieses et al. [29]; Dorjdagva et al. [30]; King et al. [31]; Siegel et al. [32]) and others refer to them simply as the concentration index (e.g., Hudson et al. [33]; Mosquera et al. [34]). Both practices give the erroneous impression that these alternative indices retain the properties and therefore normative assumptions underlying the standard CI. Moreover, several studies compare estimates of SES-related health inequality from these alternative indices to estimates from the standard CI, either within the same study or with reference to other studies in the literature. Quantitative comparisons of estimates of SES-related health inequality across indices should not be made.

#### Doubly bounded variables and the mirror property

For doubly bounded health variables (i.e., Health Utilities Index, presence or absence of an illness), some indices are sensitive to whether health is measured in terms of attainment or shortfall. That is, for the same underlying distribution of health, a relative index like the standard CI will provide a quantitatively different estimate of SES-related health inequality when inequality is measured in terms of attainment (e.g., distribution 1 in Table 2 reports 0.095 for distribution of health) rather than shortfall (e.g., distribution 4 in Table 2 reports − 0.152 for the distribution of ill-health). As a result, ranking of health distributions may also depend on the chosen perspective [4, 5]. This has led some (e.g., Erreygers et al. [5]) to argue that indices used for bivariate health inequality measurement should satisfy the “mirror property,” whereby whether a doubly bounded health variable is measured as attainment or shortfall, the index has the same magnitude but opposite sign. When the mirror property is satisfied, the ranking of distributions measured as attainment (i.e., health) will be the reverse of the ranking of distributions measured as shortfall (i.e., ill-health).^3^ In Table 2, for instance, values of the generalized concentration index, the Wagstaff index, and the Erreygers index are the same magnitude but of different sign for distributions 1 and 4.

The mirror property has important implications for the relative or absolute nature of an index. In general, absolute indices (e.g., the generalized concentration index) satisfy the mirror property, while relative indices (e.g., standard CI) satisfy the mirror property only in the special case when health and ill-health have the same mean [28, 36]. The Wagstaff and Erreygers indices also satisfy the mirror property. In fact, potential differences in estimates of inequality when inequality is assessed in terms of health attainment versus health shortfall are a more generic health measurement issue that pertains to any relative inequality measure. Preston and Taubman [37] and Deaton [38] discuss how inequality estimates depend on the chosen perspective using non-CI-based indices such as rate ratios and the Gini coefficient. These observations pertain to a longer standing issue related to relative and absolute comparisons of health outcomes, first raised, to our knowledge, by Sheps [39]).

Debate continues about whether an index satisfying the mirror property should always be used with bounded health variables. This choice likely depends on the context under study and consideration of the indices’ other properties, see Kjellsson et al. [18]. Normatively, however, choosing to use an index that satisfies the mirror property implies that the analyst chooses to view health and ill-health as equivalent.

### Changes to subgroups in the population and the weighting function

**Fig. 4:**
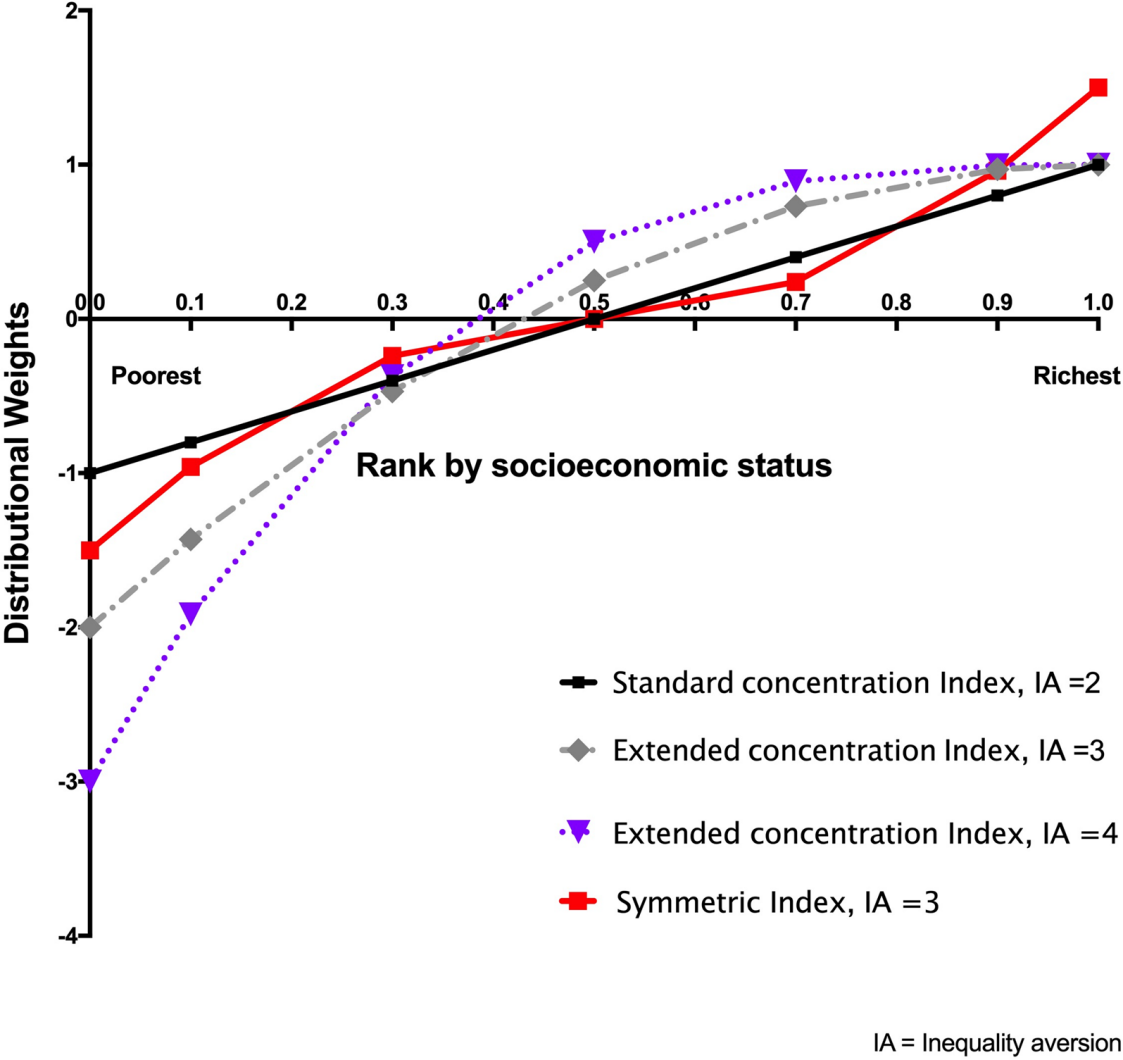
Distributional weights by rank in the socioeconomic status distribution

As discussed above, the CI-based indices are a weighted sum of *h*_*i*_, the health outcome of interest (*h*_*i*_), across all individuals in the population. The weight assigned to an individual, *z*_*i*_*,* depends on the individual’s SES rank in the population. The impact on measured SES-related inequality of changes in health among a subset of individuals in the population depends on two things: (1) the weights assigned to those individuals whose health has changed and (2) the size of the transfer. To see why, consider weighting functions for CI-based indices. Figure 4 presents weighting functions for the standard CI, the extended CI, and the symmetric index. For the standard CI, the weight assigned to the individual with the lowest SES status is − 1.0, and the weights increase linearly as rank increases, reaching a value of 0 for the median SES-ranked individual, and + 1.0 for the highest SES-ranked individual.

A key property of the extended CI is that it allows one to specify the value of the inequality aversion parameter, which reflects the assumed extent to which society dislikes inequality. Figure 4 presents the weighting function for the extended CI under two assumed values of the inequality aversion parameter (*v* = 3 and *v* = 4, where the higher value indicates stronger aversion). We can see that, compared to the standard CI, which assumes a value of *v* = 2, the difference in weights for the extended CI between poor and rich is larger, just as the values differ more when *v* = 4 than when *v* = 3. As a result, as shown in Table 2, the estimates for the extended CI with an inequality aversion parameter of 3 are consistently larger than estimates obtained for the standard CI. The nonlinear weighting functions, and in particular their concavity, reflect an aversion to SES-related inequality such that the value of the index is more sensitive to changes in health among those with lower SES than it is among those with higher SES: If the health of a rich person declines and that of a poor person improves by *the same amount*, the change in health for the poor person will receive greater weight, causing measured inequality to decrease if the distribution is pro-rich and increase if the distribution is pro-poor. (For a given health transfer, the magnitude of the change depends on the rank distance between the two participating individuals.)^4^ For this reason, quantitative comparisons of estimates of SES-related health inequality across indices with varying degrees of inequality aversion are inappropriate. The weighting function for the symmetric index follows a quite different pattern: Its shape gives greater weight to individuals at both ends of the SES distribution in a symmetric manner. For the symmetric index, what matters is how far a person is from the center of the SES distribution, so, for instance, a person with low income rank and good health does not count any more than does a person with a high income rank but poor health [5]. In viewing deviations from equality among the rich and the poor equally, the symmetric index is an alternative way of looking at systematic associations between SES and health. By focusing on the health levels at the top and bottom of the SES distribution, and giving them equal weight, the symmetric index is similar to the range and the ratio of deciles (e.g., 90/10), two measures commonly used in the epidemiological literature on health inequalities.

#### Transfers

The effect on inequality of transfers of the health outcome of interest among individuals in the population has been a central issue in inequality measurement. The notion of transfers among individuals is natural for income, and a key axiom underlying income inequality measurement has been the transfer principle [23, 41, 42]: a transfer of income from a richer person to a poorer person decreases income inequality [26, 43]. By construction, all univariate rank-dependent indices satisfy this transfer principle, and the extent to which transfers reduce inequality depends on the weighting function. The issue of transfers is more complicated in the context of the measurement of SES-related health inequality [44, 45]. In this bivariate context, the CI-based indices all conform to the principle of income-related health transfers, which holds that transfers of health from someone who is better-off in terms of socioeconomic status to someone who is worse-off decreases SES-related health inequality. The principle of income-related health transfers is the most defining normative axiom underlying all the CI-based indices.

Unlike income, health cannot be directly transferred between two people. It is possible, however, to “transfer” health among groups over time by selective investments in improving health for certain groups of the population. Hence, considerations of transfers are relevant to health distributions; however, the transfer principle raises new ethical issues for bivariate SES health inequality measurement. A transfer of health from a richer person to a poorer person may be objectionable if the richer person is in bad health and the poorer person is in good health [44, 45]. Indeed, the limited empirical evidence available indicates that the public may not support transfers of health from someone who is better-off in terms of socioeconomic status to someone who is worse-off [46]. Similarly, society may also object to policies that lower the health of some people to create a more equal distribution, a view illustrated in how some empirical papers interpret their index estimates. Walsh and Cullinan [47], for example, state “a redistribution of approximately 16.8% of the obesity rate from the poorest half of the income distribution to the richest half would result in perfect equality in the prevalence of childhood obesity,” and then claim, “Obviously a reduction in the overall rate of obesity, rather than this redistribution, would be preferable.” (p. 67).

The relevance of the transfer principle in the bivariate health-SES context is therefore contestable if health transfers are considered ethically objectionable. This may contrast with the empirical evidence on income inequality that indicates some support from the public for income transfers from the rich to the poor [26, 48]. It is unclear what the transfer principle underlying the CI-based indices should be replaced with as there is no clear alternative assumption.

## What do the estimates of inequality mean and when are they of policy concern?

In this section, we consider two main issues: (1) that indices encompass information about an entire distribution in a single number and (2) the challenge of determining if index estimates are large and of policy significance. As has been emphasized, the CI-based indices differ in important ways, which can make it challenging to interpret and compare estimates of income-related health inequality, especially across indices. For example, the underlying scale on which income-related health inequality is measured can differ across alternative CI-based indices. Further, one must be careful when interpreting the magnitude of an index estimate: A low estimate of SES-related health inequality can be generated by very little SES-related health inequality, or by large but off-setting SES-related inequality at different parts of the SES distribution. This can be revealed only by examining the concentration curve(s).^5^ In the latter case, the concentration curves will cross the diagonal one or more times. Less than half (19 of 44) of the empirical papers we reviewed presented concentration curves. Empirical examples of concentration curves crossing can be found in Buisman and García-Gómez [49] and Mosquera et al. [34]. Analogously, identical estimates of SES-related inequalities across jurisdictions (or time) can be generated by very different patterns of inequality that have different policy implications. Hence, it is important to examine the concentration curves themselves as part of interpreting the meaning and policy significance of the estimates obtained from the CI-based indices.

The absence of a natural scale for the CI-based indices is a key obstacle to their interpretation, making it difficult to determine whether inequalities are large and of policy concern. This challenge was the impetus for Koolman and van Doorslaer’s [50] redistributive interpretation. Under their redistributive interpretation, multiplying the standard CI estimate by 75 provides the percentage of the health in the population that must be transferred from the richer to poorer half of the population to arrive at an index estimate of zero. This is an approximation, and while intuitive in some ways, is subject to the same challenges noted above for interpreting point estimates (i.e., when concentration curves cross the line of equality). Moreover, Koolman and van Doorslaer’s [50] redistributive interpretation is based on a very specific notion of redistribution that conforms to the weighting scheme underlying the standard CI: The interpretation applies to the standard CI only and is not valid for other variants without a reformulation.

The vast majority of studies in our review (38 out of 44) describe the estimates of the indices in terms of their direction (whether inequalities are pro-poor or pro-rich), without elaborating on the magnitudes of the index estimates. Three studies use Koolman and van Doorslaer’s [50] redistributive interpretation to describe the index estimates. However, in all three studies a variant of the CI was used rather than the standard CI. Therefore, the validity of the interpretation is unclear. Three studies presented the point estimates of SES-related health inequality alongside the feasible bounds of the index when the outcome variable was bounded, while 14 studies using bounded outcome variables and comparing inequalities across jurisdictions, time periods, or both did not provide the feasible bounds of the index. Moreover, some studies describe the magnitudes using terms like “modest,” “large,” “pronounced,” and “high” (e.g., Devkota and Upadhyay [51]; Laskowska [52]; Xu et al. [53]; Zhang et al. [54]); however, the criteria used for classifying estimates into these categories were not stated.

## Conclusion

The increased policy focus on health inequalities requires that we have measurement tools that allow us to monitor differences and changes in socioeconomic-related health inequalities consistently and accurately (see, for example, Canadian Institute for Health Information [55]; OECD [56]). However, the different estimates of SES-related health inequality obtained by the CI-based indices reflect different normative judgments about SES-related health inequality. Notions of accuracy in terms of how well the indices reflect the extent of SES-related health inequality present in a distribution(s) are therefore conditional on a series of value-laden assumptions. The diversity in the potential conclusions and the inconsistencies in ranking are not necessarily “incorrect,” but reflect alternative perspectives of SES-related health inequality. To the extent possible, analysts should choose measure(s) that adequately capture the values of society. If this is not feasible, these normative assumptions should be made more explicit for policy-makers so that they can determine whether the normative assumptions are appropriate for the context under study. As argued in Kjellsson et al. [18], due to the diversity in potential conclusions one can obtain from the CI-based indices, there is an increased risk that analysts present the measure that best supports their chosen conclusion. This risk is mitigated if the implicit value judgments are made explicit. An alternative approach, suggested by O’Donnell et al. [40], is to estimate all feasible indices and check whether the inequality orderings are consistent across indices. If they are consistent, then it is reassuring to know that the differing normative assumptions do not influence the ordering. In situations where orderings do vary by index, the analyst can highlight which normative assumptions are crucial to the ordering.

Figure 2 summarizes key questions an analyst should ask to make an informed choice of index. First, what are the properties of the health variable for which inequalities will be assessed? The informational content of the health variable of interest determines which of the indices *can* be used. While the measurement scale and range of health variables are technical attributes and not normative per se, they may restrict which CI-based indices give meaningful results and therefore limit the choice of normative properties to those associated with this restricted set of CI-based indices. Second, what kind of changes to the distribution of health should leave measured inequality unchanged from its initial value? Considering the invariance criteria ensures that there is a match of technical assumptions *and* normative judgments. Third, if the health variable is bounded, is the mirror property relevant for the context under study? For the mirror property, in some instances, the ill-health version of the health variable, *h*_*i*_, may be more informative to policy-makers than health attainment; for example, the socioeconomic status association between premature mortality from a specific cause (e.g., death from opioids) may be more informative in monitoring the effects of a policy than simply focusing on life expectancy. Importantly, for relative indices an equiproportionate change in attainments will not necessarily constitute an equiproportionate change in shortfalls and vice versa. And fourth, does the underlying weighting scheme adequately captures who matters and by how much? Choosing a symmetric versus an asymmetric weighting scheme will weigh members of society differently which in turn will affect the ranking of distributions of SES-related health inequality, as shown in Erreygers et al. [5]. One approach to choosing an appropriate weighting function is to choose weights based on social preferences for the context under study, for example, society’s inequality aversion preferences [57, 58].

These questions may not result in a perfect match between the health variable and index. However, these series of questions may facilitate further sensitivity tests and help provide a better understanding of the index estimates. Because no index is value-neutral, the chosen index should be consistent with the measurement process, the context of the study, and the goal of the analysis.

## Supplementary Information

**Additional file 1.** Studies using the CI-based indices published between 2015–2017.

## Data Availability

All data generated or analyzed during this study are included in this published article and its supplementary information files.

## Abbreviations

CI: Concentration index
GCI: Generalized concentration index
SES: Socioeconomic status

## Footnotes

1 When concentration curves cross, different *relative* CI-based indices can rank countries differently. However, dominance of concentration curves is uninformative about rankings in terms of absolute inequalities. Additionally, without crossing concentration curves, absolute inequalities might rank countries in a different order than suggested by the dominance finding.

2 In fact, the Wagstaff index is a mixed index and the Erreygers Index is quasi-absolute. See Erreygers and Van Ourti [13] for details.

3 Note that the property of a ranking in attainment being the same as the ranking in shortfall does not imply the mirror property (see Bosmans [35]).

4 As noted in O’Donnell et al. [40]: “The weight given to the richest individual is always + 1, while the weight given to the poorest individuals becomes more negative for higher values of *v* … for *v* ≠ 2, the individual with median income does not have a weight of 0. The individual given a weight of 0 will have a lower income than the one with the median income when *v* > 2 (p. 120).

5 As noted in footnote 1, concentration curves are only informative about relative inequalities; they say nothing about absolute inequalities.
