# Supplementary material for: When the technical is also normative: a critical assessment of measuring health inequalities using the concentration index-based indices
Source: Popul Health Metr. 2022 Dec 1;20:21. doi: 10.1186/s12963-022-00299-y (PMC9713974; doi:10.1186/s12963-022-00299-y)
Supplement: Supplementary file 1 — Additional file 1. Studies using the CI-based indices published between 2015–2017. [file 12963_2022_299_MOESM1_ESM.doc]

**Additional file 1**

**Table A1 Summary of Studies using the CI-Based Indices Published between 2015 and 2017**

| **Study** | **h_i_**  **(measurement scale)** | **Concentration curve illustrated** | **Index Used** | **Match between index and health measure?** | **Range of index considered?** | **Interpretation of Results** | **Policy prescription based on the results of the index provided?** | **Estimates decomposed** |
| --- | --- | --- | --- | --- | --- | --- | --- | --- |
| Ataguba et al^1^ | Self-assessed good health as excellent, very good, or good (dichotomous) | No | Standard concentration index | Yes | Range of index not provided. | Direction: pro-rich/ pro poor. | Yes | Yes |
| Buisman and García-Gómez^2^ | Inpatient hospital use (dichotomous) | Yes | Standard concentration index | Yes | Range of index not provided. | Direction: pro-rich/ pro poor. | Yes | Yes |
| Cabieses et al^3^ | Self-reported health status (dichotomous) | Yes | Erreygers index (referred as corrected CI); Standard concentration index (to compare): | Yes for Erreygers index.  No for standard CI since mean health varies for boys and girls making it difficult to compare inequalities for the standard CI since the range of the index would vary. | Range of index not provided. | Direction: pro-rich/ pro poor. Noted: “Since concentration indices can be hard for policy makers to interpret, we also perform a simpler analysis based on more intuitive ratio and pag measures of inequality” | No | Yes |
| Capurro et al^4^ | Presence of untreated caries (dichotomous) | Yes | Standard concentration index as relative measure; Slope index of inequality for absolute measure | No  Mean health varies across the time periods examined making it difficult to interpret changes in inequalities since the range of the index would vary. | Range of index not provided. | As percentage increase in inequality. | No | No |
| Devaux^5^ | General practitioner visits (dichotomous); Specialist visits (dichotomous); Dentist visits (dichotomous); Breast and cervical cancer screening (dichotomous) | No | Wagstaff index | Yes | Yes, Wagstaff index used to accommodate bounded outcome variable. Authors awknowledge varying range when mean health varies. | Direction: pro-rich/ pro poor. | No | No |
| Devkota and Upadhyay^6^ | Probability of physician visit (dichotomous); Number of physician visits (ratio scale) | No | Standard concentration index | No  Mean health varies across different the countries for probability of physician visit making it difficult to interpret changes in inequalities since the range of the index would vary. | Range of index not provided. | Direction: pro-rich/ pro poor. | Yes | Yes |
| Dorjdagva et al^7^ | Self-assessed presence of a chronic illness (dichotomous);  Self-assessed presence of a physical disability (dichotomous) | No | Erreygers index (referred as correction to CI) | Yes | Yes, Erreygers index used to accommodate bounded outcome variable. Authors awknowledge varying range when mean health varies.   Range of index not provided. | Direction: pro-rich/ pro poor. | Yes | Yes |
| Hudson et al^8^ | Smoking status (dichotomous);  Frequent alcohol consumption defined as 5-7 days per week (dichotomous); Low physical activity (dichotomous) | No | Erreygers index (labelled as concentration index); | Yes | Yes, Erreygers index used to accommodate bounded outcome variable. Authors awknowledge varying range when mean health varies.   Range of index not provided. | Interpret using Koolman and van Doorslaer (2004), interpretation on how much to be transferred to poorest half. Unclear whether this interpretation is appropriate for Erreygers index. | No | Yes |
| Hwang et al^9^ | Self- reported visual impairments among those with diabetes (dichotomous) | Yes | Standard concentration index | Yes | Range of index not provided. | Direction: pro-rich/ pro poor. | Yes | Yes |
| Joe et al^10^ | Reporting health care utilization (dichotomous) | Yes | Standard concentration index | Yes | Range of index not provided. | Direction: pro-rich/ pro poor. | Yes | Yes |
| King et al^11^ | Avoidable mortality (dichotomous); Amenable mortality (dichotomous); Preventable mortality (dichotomous) | No | Erreygers index (Referred to as corrected CI); Standard concentration index | No for standard CI.  Mean health varies across the time periods examined making it difficult to interpret changes in inequalities for the standard CI since the range of the index would vary. | Yes, Erreygers index used to accommodate bounded outcome variable. Authors awknowledge varying range when mean health varies.   Range of index not provided. | Direction: pro-rich/ pro poor. | Yes | No |
| Laskowska^12^ | Use of medical service (dichotomous); Visits to a general practitioner (dichotomous); Visits to specialist (dichotomous); Hospital stay (dichotomous) | No | Standard concentration index | No  Mean health varies across the different regions examined making it difficult to interpret changes in inequalities since the range of the index would vary. | Range of index not provided. | Direction: pro-rich/ pro poor. | No | No |
| Layte and Nolan^13^ | General practitioner utilization (dichotomous);  Number of general practitioner visits (ratio scale); Birth weight (ratio-scale); Gestation (ratio-scale); Parental-assessed health (dichotomous); Presence of an accident (dichotomous) | No | Standard concentration index (for ratio scale variables); Erreygers index (for dichotomous variables) | Yes | Yes, Erreygers index used to accommodate bounded outcome variable. Authors awknowledge varying range when mean health varies.   Range of index not provided. | Direction: pro-rich/ pro poor. Results from the different indices compared as if equivalent. | No | Yes |
| Pal^14^ | Receipt of full antenatal care (dichotomous); Institutional or home delivery attended by skilled health professional (dichotomous) | Yes | Standard concentration index | Yes | Range of index not provided. | Not provided focus on interpreting decomposition analysis | Yes | Yes |
| Peres et al^15^ | Inadequate dentition defined as fewer than 21 natural teeth (dichotomous) | Yes | Standard concentration index; Generalized concentration index | No Mean health varies across the time period and countries examined making it difficult to interpret changes in inequalities for the standard CI since the range of the index would vary. | Range of index not provided. | Whether inequalities increased or decreased over time | No | No |
| Raittio et al^16^ | Toothache or oral discomfort (dichotomous);  Perceived current need for dental care (dichotomous); Self reported oral health status (dichotomous) | No | Standard concentration index (as relative measure); Erreygers index (as absolute measure) | No Mean health varies across the time periods examined making it difficult to compare inequalities for the standard CI since the range of the index would vary. | Range of index not provided. | Direction: pro-rich/ pro poor. | No | Yes |
| Siegel et al^17^ | BMI 30 or higher (dichotomous); Self-reported hypertension (dichotomous); Self-reported diabetes (dichotomous) | No | Wagstaff index (referred to as corrected CI) | Yes | Yes, Wagstaff index used to accommodate bounded outcome variable. Authors awknowledge varying range when mean health varies. | Direction: pro-rich/ pro poor. | No | No |
| Walsh and Cullinan^18^ | Obese (dichotomous); Overweight (dichotomous) | Yes | Wagstaff index | Yes | Yes, Wagstaff index used to accommodate bounded outcome variable. Authors awknowledge varying range when mean health varies. | Interpret using Koolman and van Doorslaer (2004), interpretation on how much to be transferred to poorest half. Unclear whether this interpretation is appropriate for Wagstaff index. Indicates that inequalities are large. | No | Yes |
| Xu et al^19^ | Whether household incurred catastrophic health expenditure (dichotomous) | Yes | Standard concentration index | No Mean health varies across the time periods examined making it difficult to interpret changes in inequalities for the standard CI since the range of the index would vary. | Range of index not provided. | Direction: pro-rich/ pro poor. | Yes | Yes |
| Zhang et al^20^ | Doctor visits (dichotomous); Inpatient care (dichotomous) | No | Standard concentration index | Yes | Range of index not provided. | Direction: pro-rich/ pro poor. | Yes | Yes |
| Carrieri and Jones^21^ | Use of e-cigs and other nicotine delivery systems (dichotomous) | No | Erreygers index | Yes | Yes  Magnitudes described relative to bounds of index (i.e. range of index) | Direction: pro-rich/ pro poor. | No | Yes |
| Davillas and Benzeval^22^ | BMI (ratio-scale, bounded); BMI components- total body fat (ratio-scale, bounded); BMI components- fat free mass (ratio-scale, bounded); Percent body fat (ratio-scale); Waist circumference (ratio-scale, bounded); Obesity based on BMI where BMI >30 (dichotomous); Where percent body fat > 25 males and >32 females (dichotomous); Obese Gallanger-percent body fat (dichotomous); Abdominal obesity where waist circumference greater than 102 cm males and 88 cm females (dichotomous) | No | Erreygers index; Wagstaff index (sensitivity analysis) | Yes | Yes, Erreygers and Wagstaff index used to accommodate bounded outcome variable. Authors awknowledge varying range when mean health varies.   Range of index not provided for Erreygers | Direction: pro-rich/ pro poor. | No | Yes |
| Gonzalo-almorox and Urbanos-garrido^23^ | No physical activity versus some physical activity (dichotomous) | No | Erreygers index; Standard concentration index | Yes for Erreygers index.  No for standard CI since mean health varies for boys and girls making it difficult to compare inequalities for the standard CI since the range of the index would vary.   Range of index not provided | Yes, Erreygers index used to accommodate bounded outcome variable. Authors awknowledge varying range when mean health varies.   Range of index not provided. | Direction: pro-rich/ pro poor . | Yes | Yes |
| Kim and Hwang^24^ | Self report gastric/colorectal cancer screening services (dichotomous) | No | Wagstaff index (referred to as renormalization of standard CI) | Yes | Yes, Wagstaff index used to accommodate bounded outcome variable. Authors awknowledge varying range when mean health varies. | Direction: pro-rich/ pro poor. | Yes | Yes |
| Ma et al^25^ | Whether household incurred catastrophic health expenditure (dichotomous) | No | Standard concentration index | No Mean health varies across the three provinces examined making it difficult to compare inequalities for the standard CI since the range of the index would vary. | Range of index not provided | Discussed trends in standard concentration index | Yes | No |
| Mosquera et al^26^ | CVD events defined as first time hospitalizations with main diagnosis of circulatory diagnosis (dichotomous) | Yes | Wagstaff index (Referred to as concentration index) | Yes | Yes, Wagstaff index used to accommodate bounded outcome variable. Authors awknowledge varying range when mean health varies. | Direction: pro-rich/ pro poor. In relation to concentration curve. | No | Yes |
| Mullachery et al^27^ | Self reported doctor visits (dichotomous); Dentist visits (dichotomous); Hospital admission (dichotomous); Reporting of usual source of care (dichotomous) | No | Standard concentration index | Yes | Yes  bounds calculated but incorrectly (p-1 and 1-p where p is prevalence) | Direction: pro-rich/ pro poor and as a percentage of feasible upper bound | No | Yes |
| Murakami and Hashimoto^28^ | Self reported dental care use (dichotomous) | No | Standard concentration index | Yes | Range of index not provided | Direction: pro-rich/ pro poor. | Yes |  |
| Palafox et al^29^ | Hypertension treatment (dichotomous); Hypertension awareness (dichotomous); Hypertension control (dichotomous) | No | Wagstaff index | Yes | Yes, Wagstaff index used to accommodate bounded outcome variable. Authors awknowledge varying range when mean health varies. | Direction: pro-rich/ pro poor. | No | No |
| Shao et al^30^ | Ill-health score constructed using self-rated health (unclear) | Yes | Standard concentration index | Unclear | Range of index not provided. | Direction: pro-rich/ pro poor. | Yes | Yes |
| Sözmen and Ünal^31^ | General practitioner visits (dichotomous); Specialist visit (dichotomous); Inpatient care (dichotomous); Dental care (dichotomous); Emergency care (dichotomous); Number of general practitioner visits (ratio scale); Number of specialist practitioner visits (ratio scale); Number of inpatient visits (ratio scale); Number of denal visits (ratio scale); Number of emergency visits (ratio scale); | Yes | Standard concentration index (for ratio scale variables); Wagstaff index (for dichotomous variables) | Yes | Yes, Wagstaff index used to accommodate bounded outcome variable. Authors awknowledge varying range when mean health varies. | Direction: pro-rich/ pro poor.  Compared estimates of standard CI to Wagstaff index as if they are equivalent. | No | Yes |
| Walsh et al^32^ | Up to date vaccination uptake (dichotomous); Vaccination usage –diphtheria-tetanus, acellular pertussis, measles-mumps-rubella and polio (dichotomous) | Yes | Wagstaff index | Yes | Yes, Wagstaff index used to accommodate bounded outcome variable. Authors awknowledge varying range when mean health varies. | Direction: pro-rich/ pro poor. Also provide Koolman and van Doorslaer (2004), interpretation on how much to be transferred to poorest half. Unclear whether this interpretation is appropriate for Wagstaff index. | No | No |
| Xu et al^33^ | Presence of depressive symptoms (dichotomous) | Yes | Standard concentration index | Yes | Range of index not provided. | Direction: pro-rich/ pro poor. | Yes | Yes |
| Zhang et al^34^ | Mortality rate reduction (ratio-scale bounded) | Yes | Standard concentration index | No Mean health varies across the time periods examined making it difficult to interpret changes in inequalities for the standard CI since the range of the index would vary. | Range of index not provided. | Direction: pro-rich/ pro poor. | Yes | No |
| Amroussia et al^35^ | Mental well-being- good versus poor mental health (dichotomous) | Yes | Wagstaff index | Yes | Yes, Wagstaff index used to accommodate bounded outcome variable. Authors acknowledge varying range when mean health varies. | Direction: pro-rich/ pro poor. | No | Yes |
| Ásgeirsdóttir and Jóhannsdóttir^36^ | Eye disease (dichotomous); Irritable bowel syndrome (dichotomous);  Chronic fatigue syndrome (dichotomous);  Cold/flu (dichotomous);  Alcoholism (or substance addiction) (dichotomous); Chronic anxiety (dichotomous); Chronic depression (dichotomous);  Anxiety (dichotomous); Serious worries (dichotomous); Sleeping difficulties (dichotomous); other mental disorders (dichotomous); Shortness of breath (dichotomous);  Debility (dichotomous); Myalgia (dichotomous); Back/shoulder pain (dichotomous); Arm pain (dichotomous); Leg pain (dichotomous); Frequent headaches (dichotomous); Toothache (dichotomous);  Abdominal pain (dichotomous);  Rheumatoid arthritis (dichotomous); Osteoarthritis (dichotomous);  Fibromyalgia (dichotomous);  Chronic back syndrome (dichotomous);  Chronic throat disease (dichotomous);  Diabetes (dichotomous); Serious headaches (dichotomous); Urinary incontinence (dichotomous); Thyroid disease (dichotomous); High blood pressure (dichotomous) | No | Wagstaff index | Yes | Yes, Wagstaff index used to accommodate bounded outcome variable. Authors awknowledge varying range when mean health varies. | Direction: pro-rich/ pro poor. | No | Yes |
| Berke-Berga et al^37^ | Self-assessed good health (dichotomous) | No | Standard concentration index | No Mean health varies across the time periods examined making it difficult to interpret changes in inequalities for the standard CI since the range of the index would vary. | Range of index not provided. | Direction: pro-rich/ pro poor. (referred to as better off and worst off) | No | Yes |
| Bilger et al^38^ | Status- obese or not (dichotomous); Depth - average excess BMI over the obesity threshold (bounded, ratio-scale);  Severity of obesity average squared excess (ratio scale) | No | Standard concentration index | No Mean health varies across the time periods examined making it difficult to interpret changes in inequalities for the standard CI for the bounded variables since the range of the index would vary. | Range of index not provided. | Direction: pro-rich/ pro poor. | No | Yes |
| Li et al^39^ | Probability of outpatient visits in the last month (dichotomous);  Total number of outpatient visits in the last month (ratio-scale); Probability of inpatient visits in the last year (dichotomous); Total number of inpatient visits in the last year (ratio scaled). | No | Standard concentration index | Yes | Range of index not provided. | Direction: pro-rich/ pro poor | Yes | Yes |
| Martenies et al^40^ | Exposure concentrations: PM_2.5_, O_3_, SO_2_, NO_2_ (ratio-scale) | No | Standard concentration index | Yes | Range of index not provided. | Direction: less socially advantaged versus more socially advantaged. | No | No |
| Mukong et al^41^ | Diagnosed with tuberculosis (dichotomous); Diagnosed with high blood pressure (dichotomous); Diagnosed with stroke (dichotomous); Diagnosed with diabetes (dichotomous); Diagnosed with stroke (dichotomous); Diagnosed with heart problems (dichotomous);  Diagnosed with cancer (dichotomous); Persistent cough (dichotomous);  Experiencing depression (dichotomous); Experiencing chest pain (dichotomous); Using PCA reduced to a single index value for health status (measurement scale unclear); Sensitivity analysis with self-assessed health (dichotomous) | No | Erreygers index | Yes | Yes, Erreygers index used to accommodate bounded outcome variable. Authors awknowledge varying range when mean health varies.   Range of index not provided. | Direction: pro-rich/ pro poor. | Yes | Yes |
| Quintal and Oliveira^42^ | Overweight or obese (dichotomous) | Yes | Wagstaff index | Yes | Yes, Wagstaff index used to accommodate bounded outcome variable. Authors awknowledge varying range when mean health varies. | Direction: pro-rich/ pro poor. | No | No |
| Si et al^43^ | Whether household incurred catastrophic health expenditure (dichotomous) | Yes | Standard concentration index | No Mean health varies across the sub groups examined making it difficult to interpret differences in inequalities since the range of the standard CI would vary. | Range of index not provided. | Direction: pro-rich/ pro poor. | Yes | Yes |
| Zheng et al^44^ | Hepatitis B awareness ordinal, score ranging from 0-5 (measurement scale unclear) | Yes | Standard concentration index | No | Range of index not provided. | Direction: pro-rich/ pro poor. | Yes | Yes |

References

1. Ataguba JE-O, Day C, Mcintyre D. Explaining the role of the social determinants of health on health inequality in South Africa. *Glob Health Action*. 2015;8:1-11.

2. Buisman LR, García-Gómez P. Inequity in inpatient healthcare utilisation 10 years after Apartheid. *Dev South Afr*. 2015;32(2):193-208.

3. Cabieses B, Cookson R, Espinoza M, Santorelli G. Did Socioeconomic Inequality in Self- Reported Health in Chile Fall after the Equity- Based Healthcare Reform of 2005? A Concentration Index Decomposition Analysis. *PLoS One*. 2015:1-21.

4. Capurro DA, Iafolla T, Kingman A, Chattopadhyay A, Garcia I. Trends in income-related inequality in untreated caries among children in the United States: findings from NHANES I, NHANES III, and NHANES 1999-2004. *Community Dent Oral Epidemiol*. 2015;43:500-510.

5. Devaux M. Income-related inequalities and inequities in health care services utilisation in 18 selected OECD countries. *Eur J Heal Econ*. 2015;16:21-33.

6. Devkota S, Upadhyay M. How do income and education affect health inequality: evidence from four developing countries. *Appl Econ*. 2015;47(52):5583-5599.

7. Dorjdagva J, Batbaatar E, Dorjsuren B, Kauhanen J. Explaining differences in education-related inequalities in health between urban and rural areas in Mongolia. *Int J Equity Health*. 2015;14:1-12.

8. Hudson E, Madden D, Mosca I. A Formal Investigation of Inequalities in Health Behaviours After Age 50 on the Island of Ireland. *Econ Soc Rev (Irel)*. 2015;46(2):233-265.

9. Hwang J, Rudnisky C, Bowen S, Johnson JA. Income-related inequalities in visual impairment and eye screening services in patients with type 2 diabetes. *J Public Health (Bangkok)*. 2015;38(4):571-579.

10. Joe W, Rudra S, Subramanian S. Horizontal Inequity in Elderly Health Care Utilization: Evidence from India. *J Korean Med Sci*. 2015;30:155-166.

11. King JM, Vallejo-Torres L, Morris S. Income related inequalities in avoidable mortality in Norway: A population-based study using data from 1994 – 2011. *Health Policy (New York)*. 2015;119:889-898.

12. Laskowska I. Availability of health services vs. health condition of residents of rural areas in Poland – Analysis performed on the basis of EHIS 2009. *Ann Agric Environ Med*. 2015;22(4):700-703.

13. Layte R, Nolan A. Income-related inequity in the use of GP services by children: a comparison of Ireland and Scotland. *Eur J Heal Econ*. 2015;16:489-506.

14. Pal R. Inequality In Maternal Health Care Utilisation In India: A Shapley Decomposition Analysis. *J Int Dev*. 2015;27:1141-1152.

15. Peres MA, Luzzi L, Peres KG, Sabbah W, Antunes JL, Do LG. Income-related inequalities in inadequate dentition over time in Australia, Brazil and USA adults. *Community Dent Oral Epidemiol*. 2015;(43):217-225.

16. Raittio E, Kiiskinen U, Helminen S, Aromaa A, Suominen AL. Income-related inequality and inequity in the use of dental services in Finland after a major subsidization reform. *Community Dent Oral Epidemiol*. 2015;43:240-254.

17. Siegel M, Mielck A, Maier W. Individual Income, Area Deprivation, And Health: Do Income-Related Health Inequalities Vary By Small Area Deprivation? *Health Econ*. 2015;24:1523-1530.

18. Walsh B, Cullinan J. Decomposing socioeconomic inequalities in childhood obesity: Evidence from Ireland. *Econ Hum Biol*. 2015;16:60-72.

19. Xu Y, Gao J, Zhou Z, et al. Measurement and explanation of socioeconomic inequality in catastrophic health care expenditure: evidence from the rural areas of Shaanxi Province. *BMC Health Serv Res*. 2015;15(256):1-10.

20. Zhang X, Wu Q, Shao Y, Fu W, Liu G, Coyte PC. Socioeconomic Inequities in Health Care Utilization in China. *Asia-Pacific J Public Heal*. 2015;27(4):429-438.

21. Carrieri V, Jones AM. Smoking for the poor and vaping for the rich? Distributional concerns for novel nicotine delivery systems. *Econ Lett*. 2016;149:71-74.

22. Davillas A, Benzeval M. Alternative measures to BMI: Exploring income-related inequalities in adiposity in Great Britain. *Soc Sci Med*. 2016;166:223-232.

23. Gonzalo-almorox E, Urbanos-garrido RM. Decomposing socio-economic inequalities in leisure-time physical inactivity: the case of Spanish children. *Int J Equity Health*. 2016;15(106):1-10.

24. Kim S, Hwang J. Assessment of trends in socioeconomic inequalities in cancer screening services in Korea , 1998 – 2012. *Int J Equity Health*. 2016;15(30):1-11.

25. Ma J, Xu J, Zhang Z, Wang J. New cooperative medical scheme decreased financial burden but expanded the gap of income-related inequity: evidence from three provinces in rural China. *Int J Equity Health*. 2016;15(72):1-11.

26. Mosquera PA, San M, Waenerlund A, Ivarsson A, Weinehall L, Gustafsson PE. Income-related inequalities in cardiovascular disease from mid-life to old age in a Northern Swedish cohort: A decomposition analysis. *Soc Sci Med*. 2016;149:135-144.

27. Mullachery P, Silver D, Macinko J. Changes in health care inequity in Brazil between 2008 and 2013. *Int J Equity Health*. 2016;15(140):1-12.

28. Murakami K, Hashimoto H. Wealth-related versus income-related inequalities in dental care use under universal public coverage: a panel data analysis of the Japanese Study of Aging and Retirement. *BMC Public Health*. 2016;16(24):1-8.

29. Palafox B, Mckee M, Balabanova D, et al. Wealth and cardiovascular health: a cross-sectional study of wealth-related inequalities in the awareness , treatment and control of hypertension in high-, middle- and low-income countries. *Int J Equity Health*. 2016;15(199):1-14.

30. Shao C, Meng X, Cui S, Wang J, Li C. Income-related health inequality of migrant workers in China and its decomposition: An analysis based on the 2012 China Labor-force Dynamics Survey data. *J Chinese Med Assoc*. 2016:1-7.

31. Sözmen K, Ünal B. Explaining inequalities in Health Care Utilization among Turkish adults: Findings from Health Survey 2008. *Health Policy (New York)*. 2016;120:100-110.

32. Walsh B, Doherty E, O’Neill C. Since The Start Of The Vaccines For Children Program, Uptake Has Increased, And Most Disparities have Decreased. *Health Aff*. 2016;35(2):356-364.

33. Xu Y, Yang J, Gao J, et al. Decomposing socioeconomic inequalities in depressive symptoms among the elderly in China. *BMC Public Health*. 2016;16(1214):1-9.

34. Zhang W, Chen D, Zhou H, et al. Regional health-care inequity in children’s survival in Zhejiang Province, China. *Int J Equity Health*. 2016;15(188):1-9.

35. Amroussia N, Gustafsson PE, Mosquera PA. Explaining mental health inequalities in Northern Sweden: a decomposition analysis. *Glob Health Action*. 2017;10:1-10.

36. Ásgeirsdóttir TL, Jóhannsdóttir HM. Income-related inequalities in diseases and health conditions over the business cycle. *Health Econ Rev*. 2017;7(12):2-17.

37. Berke-Berga A, Paul P, Valtonen H. Examining Health Inequalities in Latvia: A Decade of Association between Socioeconomic Position and Perceived Health Status. *Biomed Res Int*. 2017:1-10.

38. Bilger M, Kruger EJ, Finkelstein EA. Measuring Socioeconomic Inequality In Obesity: Looking Beyong the Obesity Threshold. *Health Econ*. 2017;26:1052-1066.

39. Li C, Dou L, Wang H, Jing S, Yin A. Horizontal Inequity in Health Care Utilization among the Middle-Aged and Elderly in China. *Int J Environ Res Public Health*. 2017;14(842):1-13.

40. Martenies SE, Milando CW, Williams GO, Batterman SA. Disease and Health Inequalities Attributable to Air Pollutant Exposure in Detroit, Michigan. *Int J Environ Res Public Health*. 2017;14(1243):1-24.

41. Mukong AK, Van Walbeek C, Ross H. Lifestyle and Income-related Inequality in Health in South Africa. *Int J Equity Health*. 2017;16(103):1-14.

42. Quintal C, Oliveira J. Socioeconomic inequalities in child obesity and overweight in Portugal. *Int J Soc Econ*. 2017;44(10):1377-1389.

43. Si Y, Zhou Z, Su M, Ma M, Xu Y, Heitner J. Catastrophic healthcare expenditure and its inequality for households with hypertension: evidence from the rural areas of Shaanxi Province in China. *Int J Equity Health*. 2017;16(27):1-12.

44. Zheng J, Li Q, Wang J, Zhang G, Wangen KR. Inequality in the hepatitis B awareness level in rural residents from 7 provinces in China. *Hum Vaccin Immunother*. 2017;13(5):1005-1013.

**Appendix B**

Literature review search strategy:

The purpose of the literature review was to identify empirical studies that employ the concentration-based indices to estimate socio-economic related health inequality. This was done to better understand how these indices are currently used. The indices considered include the standard concentration index, extended concentration index, generalized concentration index, modified concentration index, Wagstaff index, Erreygers Index, symmetric index, and generalized symmetric index. The peer-reviewed academic literature from electronic databases was searched between May and September 2018. The search terms included key words such as income related health inequality, socioeconomic health inequality, concentration index, Erreygers index, and Wagstaff index. The databases included Web of Science, EconLit, OVID Medline, EMBASE, CINAHL, PROQuest, and JSTOR. The search was also extended to the references cited in studies, as well as a search of studies citing key references related to methodological papers of CI-based indices. The search strategy was restricted to empirical studies published in the English language and in the year 2015, 2016, and 2017 since the purpose of the literature review was to examine how *recently* published studies use the CI-based indices.
